# Do lower antenatal blood pressure cut-offs in pregnant women with obesity identify those at greater risk of adverse maternal and perinatal outcomes? A secondary analysis of data from the UK Pregnancies Better Eating and Activity Trial (UPBEAT)

**DOI:** 10.1038/s41366-025-01803-8

**Published:** 2025-06-16

**Authors:** L. Slade, N. Syeda, HD Mistry, JN Bone, M. Wilson, M. Blackman, L. Poston, KM Godfrey, P. von Dadelszen, LA Magee, the UPBEAT Consortium, Andrew Shennan, Lucilla Poston, Annette Briley, Claire Singh, Paul Seed, Jane Sandall, Thomas Sanders, Nashita Patel, Angela Flynn, Shirlene Badger, Suzanne Barr, Bridget Holmes, Louise Goff, Clare Hunt, Judy Filmer, Jeni Fetherstone, Laura Scholtz, Hayley Tarft, Anna Lucas, Tsigerada Tekletdadik, Deborah Ricketts, Carolyn Gill, Alex Seroge Ignatian, Catherine Boylen, Funso Adegoke, Elodie Lawley, James Butler, Rahat Maitland, Matias Vieira, Dharmintra Pasupathy, Nina Khazaezadeh, Eugene Oteng-Ntim, Jill Demilew, Sile O’Connor, Yvonne Evans, Susan O’Donnell, Ari de la Llera, Georgina Gutzwiller, Linda Hagg, Stephen Robson, Ruth Bell, Louise Hayes, Tarja Kinnunen, Catherine McParlin, Nicola Miller, Alison Kimber, Jill Riches, Carly Allen, Claire Boag, Fiona Campbell, Andrea Fenn, Sarah Ritson, Alison Rennie, Robin Durkin, Gayle Gills, Roger Carr, Naveed Sattar, Scott Nelson, Therese McSorley, Hilary Alba, Kirsteen Paterson, Janet Johnston, Suzanne Clements, Maxine Fernon, Savannah Bett, Laura Rooney, Sinead Miller, Paul Welsh, Lynn Cherry, Melissa Whitworth, Natalie Patterson, Sarah Lee, Rachel Grimshaw, Christine Hughes, Jay Brown, Kim Hinshaw, Gillian Campbell, Joanne Knight, Diane Farrar, Vicky Jones, Gillian Butterfield, Jennifer Syson, Jennifer Eadle, Dawn Wood, Merane Todd, Asma Khalil, Deborah Brown, Paola Fernandez, Emma Cousins, Melody Smith, Jane Wardle, Helen Croker, Laura Broomfield, Keith Godfrey, Sian Robinson, Sarah Canadine, Lynne Greenwood, Stephanie Amiel, Catherine Nelson-Piercy, Gail Goldberg, Daghni Rajasingham, Penny Jackson, Sara Kenyon, Patrick Catalano

**Affiliations:** 1 Robinson Research Institute, The University of Adelaide, Adelaide, SA Australia; 2 Department of Obstetrics and Gynaecology, Women’s and Children’s Hospital, Adelaide, SA Australia; 3 Department of Women and Children’s Health, School of Life Course and Population Sciences, Faculty of Life Sciences and Medicine, King’s College London, London, UK; 4 British Columbia Children’s Hospital Research Institute, University of British Columbia, Vancouver, BC Canada; 5 Department of Obstetrics and Gynecology, University of British Columbia, Vancouver, BC Canada; 6 MRC Lifecourse Epidemiology Centre and NIHR Southampton Biomedical Research Centre, University of Southampton and University Hospital Southampton NHS Foundation Trust, Southampton, UK; 7 King’s College London/Guy’s and St Thomas’ NHS Foundation: Trust, London, UK; 8 King’s College Hospital, London, UK; 9 Newcastle University/Newcastle NHS Foundation Trust, Newcastle, UK; 10 Glasgow University and Greater Clyde Health Board, Glasgow, UK; 11 Central Manchester Hospitals Foundation Trust, Manchester, UK; 12 City Hospital Sunderland, Sunderland, UK; 13 Bradford Royal Infirmary, Bradford, UK; 14 St George’s NHS Trust, London, UK; 15 University College London, London, UK; 16 University of Southampton, Southampton, UK; 17 Trial Steering Committee, London, UK

**Keywords:** Body mass index, Translational research

## Abstract

**Background:**

Obesity is a major risk-factor for adverse pregnancy outcomes. While the 2017 American College of Cardiology/American Heart Association (ACC/AHA) classification of normal and abnormal blood pressure (BP) outside pregnancy has been suggested for use in pregnancy, the impact on adverse outcomes has not been examined specifically in women with obesity.

**Methods:**

The UK Pregnancies Better Eating and Activity Trial (UPBEAT) enroled women with a body mass index (BMI) ≥ 30 kg/m^2^. In secondary analyses, maximal antenatal BP was categorised by 2017 ACC/AHA criteria: ‘Normal’ BP (systolic [sBP] <120 mmHg and diastolic [dBP] <80 mmHg), ‘Elevated’ BP (sBP 120–129 mmHg and dBP <80 mmHg), ‘Stage 1 hypertension’ (sBP 130–139 mmHg and/or dBP 80-89 mmHg), and ‘Stage 2 hypertension’ (sBP ≥140 mmHg and/or dBP ≥90 mmHg, non-severe [sBP 140-159 mmHg and/or dBP 90–109 mmHg] and severe (sBP ≥160 mmHg and/or dBP ≥110 mmHg). Main outcomes were preterm birth, postpartum haemorrhage (PPH), birthweight <10th centile (small-for-gestational age, SGA), and neonatal intensive care unit (NICU) admission. Associations with adverse outcomes were adjusted for UPBEAT intervention, maternal age, booking BMI, ethnicity, parity, smoking, alcohol, and previous pre-eclampsia or gestational diabetes. Diagnostic test properties (positive and negative likelihood ratios, -LR and +LR) were assessed as individual categories (vs. ‘Normal’ BP), and as threshold values.

**Results:**

Severe ‘Stage 2 hypertension’ (vs. BP < 160/110 mmHg) was associated with PPH (RR 2.57 (1.35, 4.86)) and SGA (RR 2.52 (1.05, 6.07)) only in unadjusted analyses. No outcomes were associated with ‘Stage 1 hypertension’ or ‘Elevated BP’. All +LR were <5.0 and -LR ≥ 0.20, indicating that no BP threshold was useful as a diagnostic test to detect preterm birth, PPH, SGA, or NICU admission.

**Conclusions:**

Among pregnant women with obesity, we found no evidence that lowering the antenatal BP considered to be abnormal (from 140/90 mmHg) would assist in identifying women and babies at risk.

## Introduction

In 2017, the American College of Cardiology (ACC) and American Heart Association (AHA), redefined what constitutes an abnormal blood pressure (BP) outside pregnancy [1]. This was done in recognition of the increased cardiovascular risks associated with a BP lower than the previous threshold for hypertension. ‘Normal BP’ was defined as a systolic BP (sBP) < 120 mmHg and a diastolic BP (dBP) < 80 mmHg. ‘Elevated BP’ was defined as sBP of 120–129 mmHg with a dBP <80 mmHg. ‘Stage 1 hypertension’ was defined as sBP 130–139 mmHg and/or dBP 80–89 mmHg. The prior threshold for hypertension (sBP ≥140 mmHg and/or dBP ≥90 mmHg) was relabelled as ‘Stage 2 hypertension’.

Within the United States or elsewhere, no clinical practice guidelines in pregnancy define BP according to the 2017 ACC/AHA criteria [2–4]. However, several studies have reported an association between ‘Elevated BP’ or ‘Stage 1 hypertension’ (as for ‘Stage 2 hypertension’) and a heightened risk of adverse pregnancy outcomes [5–13]. Recent systematic reviews of these data have demonstrated that despite these associations, lowering the threshold for abnormal BP in pregnancy would not aid clinicians in identifying mothers and babies at risk [14, 15]; all included studies had a minority of women with obesity.

With rising rates of overweight and obesity, and the heightened risk of hypertensive disease in pregnant women with obesity, it is important to evaluate whether the association of 2017 ACC/AHA BP categories with adverse pregnancy outcomes may be different among pregnant women with obesity, compared with the general maternity population. BP measurement is readily available and of low cost, allowing easy risk stratification to identify women at particular risk of adverse pregnancy outcomes and bringing the potential to mitigate that risk through preventative measures and/or enhanced surveillance.

The UK Pregnancies Better Eating and Activity Trial (UPBEAT) was a large, multicentre, randomised trial of women with obesity in early pregnancy, allocated to a behavioural intervention (vs standard care), to assess the impact on obesity-related adverse pregnancy outcomes [16]. We undertook a secondary analysis of UPBEAT data, to evaluate the relationship between BP categorised by ACC/AHA BP criteria and pregnancy outcomes in women with obesity.

## Methods

### Study design and participants

The methods of UPBEAT have been published previously [17]. In brief, between March 2009 and June 2014, 1555 women were recruited with maternal age ≥16 years, singleton pregnancy at 15^+0^-18^+6^ weeks’ gestational age, and body mass index (BMI) ≥ 30 kg/m^2^; women were excluded if they were taking metformin, or had diabetes mellitus or another condition (e.g., chronic hypertension currently treated with antihypertensive therapy) that increased the risk of adverse pregnancy outcomes. One participant was excluded after enrolment in another trial.

### Intervention

Following written, informed consent, women were randomised to a complex behavioural intervention, focusing on a healthy diet and physical activity, or to standard antenatal care.

### Outcomes and data collection

Antenatal registration data (including measurement of BP and proteinuria) were recorded from clinical records. Study visits were conducted at trial entry between 15^+0^-18^+6^ and then again at 27^+0^-28^+6^, and 34^+0^-36^+6^ weeks’ gestation, including blood collection for cardiovascular markers.

BP was measured according to a standardised protocol, using the automated Microlife BP3BTo-A BP monitor (Microlife, Widnau, Switzerland) validated for use in pregnancy and pre-eclampsia [18], with an appropriately-sized cuff. The last BP before delivery was recorded from clinical records.

The co-primary outcomes were gestational diabetes mellitus (GDM) and large-for-gestational age infants of which neither differed between trial arms, although the intervention (vs. usual care) resulted in less gestational weight gain and more physical activity [16].

Outcomes were abstracted from clinical records after birth. Pre-eclampsia was defined as gestational hypertension (i.e., systolic BP [sBP] ≥140 mmHg or diastolic BP [dBP] ≥90 mmHg, measured twice, at least four hours apart at ≥20 weeks) and new-onset proteinuria (i.e., ≥300 mg/24 h, dipstick proteinuria ≥2+, or a spot urine protein:creatinine ratio ≥30 mg/mmol) [16]. The World Health Organisation (WHO) classifies obesity as a BMI ≥ 30 kg/m^2^, with obesity further subdivided into obesity class I (BMI 30–34.9 kg/m^2^), obesity class II (BMI 35–39.9 kg/m^2^) and obesity class III (BMI ≥ 40 kg/m^2^) [19].

Ethical approval for UPBEAT was granted by the NHS Research Ethics Committee (reference 09/H0802/5). All methods were performed in accordance with the relevant guidelines and regulations.

### This secondary analysis

Use of UPBEAT data for this project was approved by the UPBEAT Scientific Advisory Committee (Research Application Form reference 077). In this analysis, we included UPBEAT participants with data on BP from at least one study visit and the adverse pregnancy outcomes of interest. We sought to investigate whether adjusting BP thresholds for pregnant women with obesity would improve the prediction of adverse maternal and perinatal outcomes.

BP was classified according to the highest antenatal BP, using 2017 ACC/AHA criteria: ‘Normal’ (sBP <120 mmHg and dBP <80 mmHg). ‘Elevated’ (sBP 120–129 mmHg with dBP <80 mmHg), ‘Stage 1 hypertension’ (sBP 130–139 mmHg and/or dBP 80–89 mmHg), and ‘Stage 2 hypertension’ (sBP ≥140 mmHg and/or dBP ≥90 mmHg). ‘Stage 2 hypertension’ was further categorised into ‘non-severe’ (sBP 140-159 mmHg and/or dBP 90-109 mmHg) and ‘severe’ (sBP ≥160 mmHg and/or dBP ≥110 mmHg).

The main outcomes of interest were preterm birth (PTB), postpartum haemorrhage (PPH), birth weight <10th centile (small-for-gestational age, SGA), and neonatal intensive care unit (NICU) admission; none of these differed by trial arm in UPBEAT [16]. PTB was birth at <37^+0^ weeks’ gestation. PPH was an estimated blood loss ≥1000 mL. The following maternal core outcomes in hypertensive pregnancy were unavailable: stroke, eclampsia, blindness, pulmonary oedema, respiratory failure, hepatic haematoma or rupture, acute kidney injury or dialysis, elevated liver enzymes (aspartate or alanine transaminase >40IU/L), platelet count <100 × 10^9^/L, and intensive care unit admission. The following fetal/neonatal core variables were unavailable: respiratory morbidity.

For each trial participant, maximum antenatal BP was categorised according to the 2017 ACC-AHA criteria. Baseline demographics and past medical and obstetric history were summarised for the trial cohort overall, and according to the ACC-AHA BP categories; frequencies and percentages were used for categorical variables, and medians and interquartile ranges (25th, 75th centiles) for continuous variables.

### Statistical analysis

To assess the association between maximum BP and adverse pregnancy outcomes, we calculated adjusted risk ratios (aRR) using robust Poisson models. Confounders were: maternal age (yr), BMI (kg/m^2^), ethnicity (White British, Black, Asian and Other), parity (nulliparous/multiparous), smoking status (yes/no to ongoing in current pregnancy), alcohol use (yes/no to ongoing in current pregnancy), previous pre-eclampsia, and previous GDM; these were selected as variables related to BP level and outcomes, without being on the causal pathway.

The relationship between BP category and adverse outcomes were assessed in two ways. First, each BP category was treated as mutually exclusive from the others, and the risk ratio (RR) and 95% confidence interval (CI) [20] was calculated for each BP category relative to ‘Normal’ BP, using generalised estimating equations with a Poisson link function [21]. Second, analogous models were fit, but the lower limit of each category was treated as a BP cut off for the diagnosis of an abnormal BP, in the way that 140/90 mmHg is currently used; for example, for ‘Stage 1 hypertension’, the RR for each outcome was compared for women with sBP ≥130 mmHg and/or dBP ≥80 mmHg, and women with sBP <130 mmHg and dBP <80 mmHg. This analysis was repeated, adjusting for UPBEAT trial intervention group, maternal age, BMI at booking, ethnicity, parity, smoking status, alcohol use, previous pre-eclampsia, and previous GDM.

Also, for each new threshold for abnormal BP, diagnostic test properties were assessed, using sensitivity, specificity, and positive and negative likelihood ratios (+LR and -LR respectively). +LR was calculated as sensitivity/{1-specificity}, and -LR as {1-sensitivity}/specificity. LRs were considered diagnostically useful if +LR were ≥5.0 or -LR were <0.2 [22].

A sensitivity analysis was undertaken, restricting analyses to BP measurements taken under standardised conditions at study visits (and excluding BP measurements taken in routine clinical care, at antenatal care booking and again close to birth). We examined the impact on the association of each BP category (vs. ‘Normal’ BP) with adverse outcomes, as well as the diagnostic test properties of the BP threshold associated with each category.

All analyses were undertaken using R statistical software, with 95% confidence intervals (CIs) considered significant if they did not cross 1.0. No adjustment was made for multiple comparisons. As a secondary analysis of an existing dataset, no sample size calculation was undertaken.

## Results

**Fig. 1:**
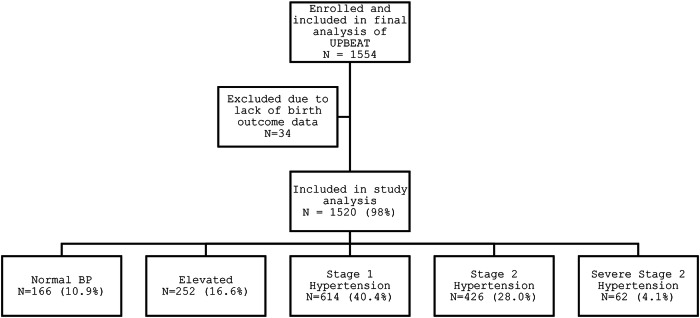
Flow diagram of participants included in this secondary analysis of data from the UPBEAT trial. *BP (blood pressure)*.

Of the 1554 women in UPBEAT, 1520 (97.8%) were included in this analysis (Fig. 1), following exclusion of 34 women with missing birth outcome data.

Table 1 (‘Overall’ column) shows that on average, UPBEAT participants were aged 30 years at booking, mostly White British, and more than half had a BMI consistent with at least class II obesity (≥35 kg/m^2^). Most women who were multiparous had a previous uncomplicated pregnancy, but family history was significant for at least one cardiovascular risk factor in almost half of women. Women had their first antenatal visit at an average of 10 weeks’ gestation, few conceived by artificial reproductive technologies, and just over 40% were nulliparous. Just over 10% were taking low-dose aspirin. Few reported being smokers or consuming alcohol. Half had been randomised to the intervention arm.

**Table 1:** Baseline characteristics overall and according to antenatal BP (mean ± SD or median [IQR] *N* women (%) unless otherwise specified).

		Antenatal BP (mmHg)^a^				
		‘Normal BP’	‘Elevated BP’	‘Stage 1 HTN’	‘Stage 2 HTN’ Non-severe	‘Stage 2 HTN’ Severe
	Overall	<120/<80	120–129/<80	130–139/80–89	≥140/≥90	≥160/≥110
	*N* = 1520	(*N* = 166, 10.9%)	(*N* = 252, 16.6%)	(*N* = 614, 40.4%)	(*N* = 426, 28.0%)	(*N* = 62, 4.1%)
Demographics						
Maternal age (yr)	30.5 ± 5.5	30.8 ± 5.5	30.5 ± 5.4	30.4 ± 5.7	30.3 ± 5.3	30.7 ± 5.4
Ethnicity						
White British	957 (63.0%)	67 (40.4%)	150 (59.5%)	390 (63.5%)	304 (71.4%)	46 (74.2%)
Black	388 (25.5%)	62 (37.3%)	70 (27.8%)	164 (26.7%)	81 (19.0%)	11 (17.7%)
Asian	91 (6.0%)	24 (14.5%)	12 (4.8%)	34 (5.5%)	18 (4.2%)	4 (6.5%)
Other	84 (5.5%)	13 (7.8%)	20 (7.9%)	26 (4.2%)	23 (5.4%)	2 (3.2%)
BMI (kg/m^2^)						
Mean (SD)	36.3 ± 4.8	34.4 ± 3.1	35.3 ± 4.1	36.3 ± 4.7	37.8 ± 5.3	37.3 ± 5.6
30–34.9	666 (43.8%)	113 (68.1%)	149 (59.1%)	302 (49.2%)	157 (36.9%)	29 (46.8%)
35–39.9	551 (36.2%)	44 (26.5%)	72 (28.6%)	200 (32.6%)	161 (37.8%)	16 (25.8%)
>40	303 (20.0%)	9 (5.4%)	31 (12.3%)	112 (18.2%)	108 (25.4%)	17 (27.4%)
Previous history						
Chronic hypertension	49 (3.3%)	0	4 (1.6%)	10 (2.3%)	31 (7.3%)	5 (8.1%)
Parous women	(*N* = 859)	(*N* = 124)	(*N* = 153)	(*N* = 349)	(*N* = 212)	(*N* = 21)
Prior pre-eclampsia	69 (8.0%)	6 (4.8%)	8 (5.2%)	20 (5.9%)	29 (13.7%)	6 (28.6%)
Prior GDM	32 (3.7%)	5 (4.0%)	5 (3.3%)	15 (3.9%)	7 (3.3%)	0
History among first degree relative(s)						
Hypertension	702 (46.2%)	81 (48.8%)	104 (41.3%)	271 (43.9%)	218 (51.2%)	28 (45.2%)
DM (Type 1 or 2)	400 (26.3%)	57 (34.3%)	56 (22.2%)	167 (26.3%)	109 (25.6%)	14 (22.6%)
Ischaemic heart disease	232 (15.3%)	23 (13.9%)	41 (16.3%)	85 (15.2%)	75 (17.6%)	8 (12.9%)
Stroke	123 (8.1%)	17 (10.2%)	25 (9.9%)	45 (7.4%)	31 (7.3%)	5 (8.1%)
Pre-eclampsia	173 (11.4%)	14 (8.4%)	25 (9.9%)	68 (11.7%)	58 (13.6%)	8 (12.9%)
Current pregnancy						
GA at booking (weeks)	10.3 ± 2.1	10.3 ± 1.8	10.0 ± 1.8	10.4 ± 2.1	10.3 ± 2.3	10.3 ± 1.9
Conceived by ART						
Ovarian stimulation	30 (2.0%)	0	5 (1.9%)	15 (2.3%)	10 (2.3%)	0
In vitro fertilisation	22 (1.4%)	1 (0.6%)	4 (1.6%)	12 (2.0%)	5 (1.1%)	0
Nulliparity	661 (43.5%)	42 (25.3%)	99 (39.3%)	266 (44.9%)	213 (50.0%)	41 (66.1%)
Aspirin at first visit	195 (12.8%)	14 (8.4%)	15 (5.9%)	67 (11.9%)	83 (19.5%)	16 (25.8%)
Cigarettes at first visit	105 (6.9%)	11 (6.6%)	20 (7.9%)	42 (7.2%)	31 (7.3%)	1 (1.6%)
Alcohol at first visit	66 (4.3%)	5 (3.0%)	12 (4.8%)	31 (5.7%)	17 (4.0%)	1 (1.6%)
UPBEAT information						
Intervention arm	763 (50.2%)	76 (45.8%)	136 (53.9%)	306 (51.9%)	211 (49.5%)	34 (54.8%)

*APH* antepartum haemorrhage, *ART* artificial reproductive technologies, *BP* blood pressure, *dBP* diastolic blood pressure, *DM* diabetes mellitus, *GDM* gestational diabetes mellitus, *PPH* postpartum haemorrhage, *sBP* systolic blood pressure.

^a^As assessed at outpatient antenatal visits or medical assessment unit visits prior to the delivery admission. BP was categorised according to the 2017 American College of Cardiology/American Heart Association criteria as follows: ‘Normal BP’ (sBP < 120 mmHg and dBP < 80 mmHg), ‘Elevated BP’ (sBP 120–129 mmHg but dBP < 80 mmHg), ‘Stage 1 hypertension’ (sBP 130–139 mmHg and/or dBP 80–89 mmHg), and ‘Stage 2 hypertension’ (sBP ≥ 140 mmHg and/or dBP ≥ 90 mmHg).

Table 1 (‘Antenatal BP’ columns) shows that few women were categorised as having ‘Normal’ BP (166, 10.9%) or ‘Elevated’ BP (252, 16.6%), with most women having ‘Stage 1 hypertension’ (614, 40.4%) or ‘Stage 2 hypertension’ (488, 32.1%; 28.0% non-severe and 4.1% severe). Many baseline and pregnancy characteristics differed according to BP category; women with ‘Normal’ BP or ‘Elevated’ BP had a lower mean BMI and were more likely to have WHO class I obesity (rather than III). Women with ‘Stage 1 hypertension’ or ‘Stage 2 hypertension’ were more likely to have a higher BMI, prior pre-eclampsia, and be on aspirin from their first antenatal visit.

Table 2 (‘Overall’ column) shows that on average, women delivered at 39 weeks’ gestation, with one-third undergoing labour induction and a similar number undergoing a Caesarean delivery. A clinical diagnosis of gestational hypertension or pre-eclampsia developed in 8.7% of women overall. There were no maternal deaths, abruption was rare, and a minority of women developed GDM or PPH. Most women delivered term, appropriately-grown babies who were not admitted to neonatal care (which was required most often for respiratory problems).

**Table 2:** Labour, delivery, and maternal and perinatal outcomes (mean ± SD or median [IQR] *N* (%) unless otherwise specified).

		Antenatal BP (mmHg)^a^				
		‘Normal BP’	‘Elevated BP’	‘Stage 1 HTN’	‘Stage 2 HTN’ Non-severe	‘Stage 2 HTN’ Severe
	Overall	<120/<80	120–129/<80	130–139/80–89	≥140/≥90	≥160/≥110
	*N* = 1520	(*N* = 166)	(*N* = 252)	(*N* = 614)	(*N* = 426)	(*N* = 62)
Labour and delivery						
GA at delivery (wk)	39 ± 2.3	39 ± 2.4	39 ± 2.4	39 ± 2.4	39 ± 2.1	38 ± 3.0
Labour induction	526 (34.6%)	36 (21.7%)	74 (29.4%)	202 (34.0%)	172 (40.4%)	42 (67.7%)
Mode of delivery						
Unassisted vaginal	798 (52.5%)	107 (64.5%)	145 (57.5%)	327 (53.3%)	196 (46.0%)	23 (37.1%)
Assisted vaginal	178 (11.7%)	13 (7.8%)	31 (12.3%)	70 (11.4%)	54 (12.7%)	10 (16.1%)
Caesarean	544 (35.8%)	46 (27.7%)	76 (30.2%)	217 (35.3%)	176 (41.3%)	29 (46.8%)
Maternal complications and co-morbidities						
Gestational hypertension	78 (5.1%)	0/153	2/245	4/597 (2.3%)	54/420 (12.7%)	18/62 (29.0%)
Pre-eclampsia	54 (3.6%)	0/153	0/247	1/603	30/424 (7.0%)	23/62 (37.1%)
Maternal death	0	0	0	0	0	0
Placental abruption	9 (0.6%)	1 (0.6%)	1 (0.4%)	2 (0.3%)	3 (0.7%)	2 (3.2%)
PPH ≥ 1000 mL	200 (13.2%)	15 (9.0%)	23 (9.1%)	78 (12.7%)	68 (15.9%)	16 (25.8%)
GDM	326 (21.4%)	21 (12.7%)	42 (16.7%)	130 (21.1%)	115 (27.0%)	18 (29.0%)
* Missing*	38 (2.5%)	12 (7.2%)	7 (2.8%)	16 (2.6%)	4 (0.9%)	0
Perinatal outcomes						
Stillbirth	10 (0.7%)	1 (0.6%)	3 (1.2%)	3 (0.5%)	2 (0.4%)	1 (1.6%)
Neonatal death	5 (0.3%)	1 (0.6%)	1 (0.4%)	0	3 (0.7%)	1 (1.6%)
*Missing*	18 (1.2%)	7 (4.2%)	5 (1.9%)	4 (0.7%)	1 (0.2%)	0
Birth at <34 weeks	36 (2.4%)	6 (3.6%)	3 (1.2%)	16 (2.6%)	7 (1.6%)	4 (6.5%)
Birth at <37 weeks	99 (6.5%)	13 (7.8%)	16 (6.3%)	36 (5.9%)	35 (8.2%)	9 (14.5%)
Gender						
Male	767 (50.2%)	84 (50.6%)	137 (54.4%)	294 (47.9%)	227 (53.3%)	25 (40.3%)
Female	752 (49.5%)	82 (49.4%)	114 (45.2%)	320 (52.0%)	199 (46.7%)	37 (59.7%)
Birthweight (g)						
<10th centile [WHO]	94 (6.2%)	10 (6.0%)	13 (5.2%)	33 (5.4%)	29 (6.8%)	9 (14.5%)
<5th centile [WHO]	43 (2.8%)	3 (1.8%)	7 (2.7%)	14 (2.3%)	14 (3.3%)	5 (22.6%)
>90th centile [WHO]	180 (11.8%)	23 (13.9%)	33 (13.1%)	67 (10.9%)	51 (12.0%)	6 (9.7%)
>95th centile [WHO]	93 (6.1%)	11 (6.6%)	15 (5.9%)	37 (6.0%)	27 (6.3%)	3 (4.8%)
Apgar score <7 at 5 min	32 (2.1%)	4 (2.4%)	7 (2.7%)	8 (1.3%)	10 (2.3%)	3 (4.8%)
*Missing*	26 (1.7%)	7 (4.2%)	5 (1.9%)	11 (1.8%)	1 (0.2%)	0
Neonatal care admission	120 (7.9%)	15 (9.0%)	14 (5.5%)	38 (6.2%)	53 (12.4%)	9 (14.5%)
>48 h	64 (4.2%)	8 (4.8%)	11 (4.4%)	27 (4.4%)	24 (5.6%)	6 (9.7%)
Mechanical ventilation	40	5	5	14	11	5

*APH* antepartum haemorrhage, *ART* artificial reproductive technologies, *BP* blood pressure, *GDM* gestational diabetes mellitus, *HDP* hypertensive disorder of pregnancy, *NA* not applicable, *PPH* postpartum haemorrhage, *SGA* small for gestational age.

^a^As assessed at outpatient antenatal visits or medical assessment unit visits prior to the delivery admission. BP was categorised according to the 2017 American College of Cardiology/American Heart Association criteria as follows: ‘Normal BP’ (sBP < 120 mmHg and dBP < 80 mmHg), ‘Elevated BP’ (sBP 120–129 mmHg but dBP < 80 mmHg), ‘Stage 1 hypertension’ (sBP 130–139 mmHg and/or dBP 80–89 mmHg), and ‘Stage 2 hypertension’ (sBP ≥ 140 mmHg and/or dBP ≥ 90 mmHg).

Table 2 (‘Antenatal BP’ columns) shows that many pregnancy characteristics differed by BP category, with higher BP associated with labour induction, instrumental vaginal birth or Caesarean section in labour, complications of gestational hypertension or PPH, and NICU admission. Placental abruption, stillbirth, and neonatal death were too infrequent to include in subsequent analyses of their relationship with BP category.

Table 3 shows that only Severe ‘Stage 2 hypertension’ was associated with higher risk of PPH (RR 2.57 (1.35, 4.86)) and SGA (RR 2.52 (1.05, 6.07), but only in unadjusted analyses. Elevated and ‘Stage 1 hypertension’ were not associated with outcomes. Adjusted and unadjusted values did not vary substantially.

**Table 3:** Relative risks by the American College of Cardiology/American Heart Association categories as thresholds for adverse pregnancy outcome.

		Antenatal BP (mmHg)^a^				
		‘Normal BP’	‘Elevated BP’	‘Stage 1 HTN’	‘Stage 2 HTN’	
					‘Stage 2 HTN’ Non-Severe	‘Stage 2 HTN’ Severe
		<120/<80	120–129/<80	130–139/80–89	140/90–160/110	≥160/≥110
Preterm birth	RR	Ref	0.75 [0.36, 1.58]	0.83 [0.45, 1.55]	0.77 [0.40, 1.50]	1.89 [0.84, 4.27]
	aRR^b^	Ref	0.74 [0.35, 1.57]	0.81 [0.43, 1.52]	0.71 [0.35, 1.41]	1.71 [0.73, 4.02]
PPH	RR	Ref	0.94 [0.50, 1.76]	1.29 [0.77, 2.17]	1.60 [0.94, 2.71]	2.57 [1.35, 4.86]
	aRR^b^	Ref	0.83 [0.45, 1.55]	1.13 [0.67, 1.90]	1.33 [0.78, 2.27]	1.91 [0.99, 3.68]
SGA	RR	Ref	0.93 [0.41, 2.13]	0.97 [0.47, 1.98]	1.19 [0.58, 2.46]	2.52 [1.05, 6.07]
	aRR^b^	Ref	0.79 [0.35, 1.79]	0.84 [0.41, 1.71]	0.99 [0.48, 2.06]	2.03 [0.81, 5.11]
NICU admission	RR	Ref	0.74 [0.37, 1.47]	0.73 [0.41, 1.31]	1.08 [0.61, 1.93]	1.62 [0.74, 3.56]
	aRR^b^	Ref	0.73 [0.37, 1.46]	0.70 [0.39, 1.27]	1.02 [0.56, 1.86]	1.53 [0.65, 3.60]

*aRR* adjusted relative risk, *BP* blood pressure, *NICU* neonatal intensive care unit, *PPH* postpartum haemorrhage, *PTB* preterm birth, *RR* relative risk, *SGA* small for gestational age.

^a^As assessed at outpatient antenatal visits or medical assessment unit visits prior to the delivery admission. BP was categorised according to the 2017 American College of Cardiology/American Heart Association criteria as follows: ‘Normal BP’ (sBP < 120 mmHg and dBP < 80 mmHg), ‘Elevated BP’ (sBP 120–129 mmHg but dBP < 80 mmHg), ‘Stage 1 hypertension’ (sBP 130–139 mmHg and/or dBP 80–89 mmHg), and ‘Stage 2 hypertension’ (sBP ≥ 140 mmHg and/or dBP ≥ 90 mmHg).

^b^The RR was adjusted for UPBEAT intervention status, maternal age, body mass index at booking, ethnicity, parity, smoking status, alcohol use, previous pre-eclampsia, and previous gestational diabetes.

Table 4 shows that only Severe ‘Stage 2 hypertension’ had a high specificity, and for all adverse outcomes. However, the sensitivity was very low (<10%). While ‘Normal’ BP had high sensitivity (>85%), specificity (<20%) was very low for the outcomes evaluated. None of the potential thresholds for abnormal BP (including the current 140/90 mmHg for ‘Stage 2 hypertension’) were useful as a diagnostic test to identify women at low or high risk of adverse outcomes (i.e., -LR ≥ 0.20 and +LR < 5.0); while this was based on point estimates, no lower limit of the 95% CI for -LR was <0.20, and no upper limit for +LR was ≥5.0.

**Table 4:** Diagnostic test properties of American College of Cardiology/American Heart Association BP categories for adverse pregnancy outcomes^a^.

Outcomes and BP thresholds	Sensitivity (95% CI)	Specificity (95% CI)	Positive LR (95% CI)	Negative LR (95% CI)
Preterm Birth				
‘Elevated BP’	0.88 (0.80, 0.94)	0.10 (0.09, 0.12)	0.98 (0.91, 1.06)	1.17 (0.67, 2.04)
‘Stage 1 HTN’	0.74 (0.64, 0.82)	0.27 (0.24, 0.29)	1.01 (0.89, 1.14)	0.98 (0.70, 1.38)
‘Non-severe ‘Stage 2 HTN’	0.34 (0.25, 0.45)	0.68 (0.65, 0.70)	1.06 (0.80, 1.41)	0.97 (0.84, 1.12)
‘Severe ‘Stage 2 HTN’	0.09 (0.04, 0.17)	0.96 (0.95, 0.97)	2.39 (1.22, 4.70)	0.95 (0.89, 1.01)
Postpartum haemorrhage				
‘Elevated BP’	0.92 (0.88, 0.96)	0.10 (0.09, 0.12)	1.03 (0.99, 1.08)	0.72 (0.43, 1.2)
‘Stage 1 HTN’	0.81 (0.75, 0.87)	0.27 (0.25, 0.30)	1.12 (1.04, 1.21)	0.68 (0.50, 0.92)
‘Non-severe ‘Stage 2 HTN’	0.42 (0.35, 0.49)	0.69 (0.66, 0.71)	1.35 (1.12, 1.61)	0.84 (0.75, 0.95)
‘Severe ‘Stage 2 HTN’	0.08 (0.05, 0.13)	0.96 (0.95, 0.97)	2.26 (1.31, 3.92)	0.95 (0.91, 0.99)
Small-for-gestational age				
‘Elevated BP’	0.90 (0.83, 0.96)	0.11 (0.09, 0.12)	1.01 (0.94, 1.08)	0.91 (0.48, 1.72)
‘Stage 1 HTN’	0.77 (0.67, 0.85)	0.27 (0.25, 0.29)	1.05 (0.93, 1.18)	0.87 (0.60, 1.27)
‘Non-severe ‘Stage 2 HTN’	0.40 (0.30, 0.51)	0.68 (0.66, 0.70)	1.27 (0.98, 1.64)	0.88 (0.74, 1.04)
‘Severe ‘Stage 2 HTN’	0.10 (0.04, 0.17)	0.96 (0.95, 0.97)	2.52 (1.29, 4.95)	0.94 (0.88, 1.00)
NICU admission				
‘Elevated BP’	0.88 (0.81, 0.93)	0.10 (0.09, 0.12)	0.99 (0.92, 1.05)	1.13 (0.67, 1.89)
‘Stage 1 HTN’	0.75 (0.66, 0.82)	0.27 (0.25, 0.29)	1.03 (0.92, 1.14)	0.93 (0.67, 1.28)
‘Non-severe ‘Stage 2 HTN’	0.42 (0.33, 0.51)	0.68 (0.66, 0.71)	1.32 (1.05, 1.65)	0.85 (0.73, 1.00)
‘Severe ‘Stage 2 HTN’	0.07 (0.03, 0.14)	0.96 (0.95, 0.97)	1.94 (0.98, 3.84)	0.96 (0.91, 1.01)

*BP* blood pressure, *CI* confidence interval, *−LR* negative likelihood ratio, *+LR* positive likelihood ratio, *NICU* neonatal intensive care unit, *PPH* postpartum haemorrhage, *SGA* small-for-gestational age.

^a^As assessed at outpatient antenatal visits or medical assessment unit visits prior to the delivery admission. BP was categorised according to the 2017 American College of Cardiology/American Heart Association criteria as follows: ‘Normal BP’ (sBP < 120 mmHg and dBP < 80 mmHg), ‘Elevated BP’ (sBP 120–129 mmHg but dBP < 80 mmHg), ‘Stage 1 hypertension’ (sBP 130–139 mmHg and/or dBP 80–89 mmHg), and ‘Stage 2 hypertension’ (sBP ≥ 140 mmHg and/or dBP ≥ 90 mmHg).

In the sensitivity analysis restricted to BP measurements collected at study visits, the results were similar to those overall, in that only Severe ‘Stage 2 hypertension’ was associated with adverse outcomes—but in this case, the association was with PTB in adjusted analyses (aRR 6.29 (1.97, 20.08)) (Table S1), and the +LR was good (9.57 (1.62, 56.60)), suggesting that risk was meaningfully increased (Table S2).

## Discussion

### Principal findings

In this population of pregnant women with obesity enrolled in a randomised trial of a diet and lifestyle intervention, our analyses found no association of the ACC/AHA BP categories with the adverse pregnancy outcomes of PPH, preterm birth, birthweight <10th centile, or NICU admission. However, in sensitivity analyses restricted to study visit BP measurements, Severe ‘Stage 2 hypertension’ was associated with PTB, and with a good +LR.

### Results in the context of what is known

Measuring BP in women with overweight and obesity, can represent challenges in standardisation, particularly when routine measurements are relied on in study settings. The ACC/AHA emphasise the importance of an appropriately sized BP cuff [1], as used in UPBEAT. Using a cuff size that is too small is likely to result in an overestimation of BP. For example, using a standard-sized cuff (vs. a larger one as dictated by arm circumference) resulted in an extra 7% of women misdiagnosed with hypertension [23]. Also, with the larger cylindrically-shaped cuffs, inaccurate BP measurements can result from an uneven fit over the distal end of the upper arm; overestimation of BP and overdiagnosis of hypertension is then more likely [24]. For this reason, troncoconical cuffs are suggested outside pregnancy for persons with a mid-arm circumference above 42 cm [24–26]. The UPBEAT trial used a standardised approach to measure BP, including an appropriate cuff size.

Several authors have reviewed the relationships of 2017 ACC/AHA BP criteria and adverse pregnancy outcomes, with varied results [14, 15]. Our findings contrast with data from large population-based studies, including women of all BMI in pregnancy [6, 27], that have demonstrated an association between higher BP category and higher risk of adverse pregnancy outcomes, including preterm birth, SGA infants, and NICU admission evaluated in our study. There was a large degree of heterogeneity in this review, particularly looking at the background risk of the women, explained by both their demographics, and their individual health characteristics.

The average BMI of women included varied across these reports, with few studies including a significant proportion of women with obesity. Of included studies that controlled for BMI in their analyses, and reported both unadjusted and adjusted analyses, adjustment reduced the strength of association between BP categories and adverse pregnancy outcome [6–13, 28–34]. However, all adjusted for multiple confounders that included BMI but also commonly maternal age, parity, and race/ethnicity. Of studies that reported only adjusted analyses, none adjusted only for BMI.

The pattern of associations seen consistently throughout these studies was not seen in this analysis restricted only to women with obesity. This could be influenced by the misclassification of women because of difficulty in accurately measuring BP in obesity, or perhaps masked by the multifactorial interaction of obesity with the pathophysiology of adverse pregnancy outcomes [35]. However, this analysis confirms that a different BP threshold need not be applied for women with obesity, despite the difficulties in accurate BP measurement and complex interplay of risk factors in this high-risk population.

Of note, our sensitivity analysis restricted to study visit BP measurements confirmed the importance of Severe ‘Stage 2 hypertension as a risk factor for adverse outcomes.

Wu reported the pregnancy outcomes of 47,874 women with a singleton pregnancy, who had BP in pregnancy of less than 140/90 mmHg in early pregnancy [34]. They also excluded women with multiple pregnancy, stillbirth, renal disease, pre-existing diabetes and thyroid dysfunction, leaving a relatively low-risk cohort of women. Approximately a quarter of these women were multiparous, and although they presented individual analyses by BMI category, the highest category was a BMI of at least 25 kg/m^2^, which accounted for only 14% of their cohort. Within this group, using blood pressures from <20 weeks in the pregnancy, an association was seen for the hypertensive disorders with ‘Stage 1 hypertension’. The other pregnancy outcomes seen in this study were not reported on by BMI category. Similar findings were observed by Hauspurg, in a secondary analysis of the Nulliparous Pregnancy Outcome Study: Monitoring Mothers-to-be [8]. Based on BP in early pregnancy, and including a larger proportion of women with overweight and obesity, an association was again seen. No other pregnancy outcomes were stratified by BMI.

Obesity is an important risk factor for both hypertension and cardiovascular disease outside pregnancy, as well as hypertension and many adverse pregnancy outcomes [36]. It is likely that this is through similar mechanisms by which obesity influences the risk of metabolic syndrome and cardiovascular disease outside pregnancy [35]. The contribution of adipose tissue to insulin resistance, cytokine release and inflammation are all proposed contributors to the link between obesity and pre-eclampsia, in the same way that obesity is linked to cardiovascular disease outside pregnancy [35, 37]. There are also multiple associations between the dietary and lifestyle factors that contribute to obesity and pre-eclampsia, including low rates of physical activity, and a sub-optimal diet, including a high intake of refined sugars [35].

### Clinical implications

BP thresholds below the current 140/90 mmHg did not identify women at increased risk of adverse outcomes in pregnancy complicated by obesity. While our findings confirm the importance of severe ‘Stage 2 hypertension’, they do not support a lowering of BP thresholds in pregnant women with obesity to identify those at increased risk of adverse pregnancy outcomes.

### Research implications

This analysis has shown that the relationship between BP and adverse pregnancy outcome is different in a cohort of only women with obesity, compared with previous observations in cohorts of pregnant women with heterogeneous BMI. Future work should aim to evaluate, as much as possible, the complex interplay between risk factors for adverse pregnancy outcomes and the associations and contributors to overweight and obesity.

### Strengths and limitations

Categorisation of BP was based on the highest BP measurement available throughout the pregnancy. However, in the UPBEAT trial, nearly 20% of values were missing for the third study visit, which is the latest in gestation. Given that the expected trajectory of BP throughout pregnancy is a gradual rise throughout third trimester, this could have led to the misclassification into a lower BP category and overestimation of any BP category-outcome effects. The BP values used at the first antenatal appointment and at last appointment were also from clinical care, not from the standardised measurements, done as part of the trial; however, sensitivity analyses excluding them produced similar results for all but severe ‘Stage 2 hypertension’ for which the association with adverse outcome was strengthened. Detailed information about BP medication was not available at each visit, however given that BP was classified according to the maximum measurement, this is unlikely to have changed groupings; UK guidelines until 2019 did not recommend treatment of BP until 150/100 mmHg, so it is unlikely that treatment has changed classification of these women. Treated hypertension should overestimate the treatment effect for BP thresholds below Stage 2 hypertension. Also, the incidence of preeclampsia in this cohort was low given that all women had obesity. This may have related to the fact that before 2019, the UK used only a restrictive definition of pre-eclampsia (based on only hypertension and new-onset proteinuria), trial participants were more likely to have BP measured properly with the appropriately-sized cuff, and as hypertension was not a focus of UPBEAT, a medical record diagnosis of pre-eclampsia (which is less reliable) was used; of note, while the UPBEAT lifestyle intervention (vs. usual care) did result in reduced gestational weight gain and increased physical activity, there was no between-group difference in pre-eclampsia (and gestational hypertension was not reported). The cause of preterm birth (iatrogenic or spontaneous) was not available. We were limited by the information recorded in the original trial, which did not include indication for caesarean section or the number of antenatal visits recorded.

## Conclusions

This analysis has not demonstrated that lowering the BP value considered to be abnormal (from 140/90 mmHg) in women with obesity would assist in identifying women and babies at risk.

## Supplementary information

Supplementary Appendix

## Data Availability

The datasets analysed during the current study are available in from the UPBEAT Consortium. https://www.medscinet.net/upbeat/default.aspx.
