# Supplementary material for: Do lower antenatal blood pressure cut-offs in pregnant women with obesity identify those at greater risk of adverse maternal and perinatal outcomes? A secondary analysis of data from the UK Pregnancies Better Eating and Activity Trial (UPBEAT)
Source: Int J Obes (Lond). 2025 Jun 16;49(8):1523–31. doi: 10.1038/s41366-025-01803-8 (PMC12396956; doi:10.1038/s41366-025-01803-8)
Supplement: Supplementary file 1 — Supplementary Appendix [file 41366_2025_1803_MOESM1_ESM.docx]

**SUPPLEMENTARY APPENDIX**

| **Table** | **Title** | **Page number** |
| --- | --- | --- |
| Table S1 | Sensitivity analysis restricted to study visit BP measurements: relative risks and 95% confidence intervals for adverse pregnancy outcomes, according to ACC/AHA BP categories as thresholds for abnormal BP | 1 |
| Table S2 | Sensitivity analysis restricted to study visit BP measurements: diagnostic test properties of ACC/AHA BP categories for detection of adverse pregnancy outcomes, according to ACC/AHA BP categories as thresholds for abnormal BP | 2 |

**Table S1:** Sensitivity analysis restricted to study visit BP measurements: relative risks and 95% confidence intervals for adverse pregnancy outcomes, according to ACC/AHA BP categories as thresholds for abnormal BP

| **Outcomes** |  | **‘Normal BP’**  (N=166) | **‘Elevated BP’**  (N=252) | **‘Stage 1**  **hypertension’**  (N=614) | **‘Stage 2 hypertension’**  (N=488) | **Severe ‘Stage 2 hypertension’**  (N=62) |
| --- | --- | --- | --- | --- | --- | --- |
| PPH | aRRł | Ref | 0.87 [0.59, 1.29] | 1.21 [0.88, 1.67] | 1.11 [0.72, 1.73] | NAǁ |
|  | aRR2ǂ | Ref | 0.86 [0.58, 1.28] | 1.23 [0.89, 1.70] | 1.14 [0.73, 1.77] | NAǁ |
| Preterm birth | aRRł | Ref | 1.19 [0.70, 2.03] | 1.03 [0.63, 1.68] | 1.02 [0.52, 1.99] | 6.29 [1.97, 20.08] |
|  | aRR2ǂ | Ref | 1.22 [0.71, 2.08] | 1.11 [0.68, 1.81] | 1.10 [0.57, 2.15] | 6.70 [2.33, 19.21] |
| Birthweight <10^th^ centile | aRRł | Ref | 0.74 [0.40, 1.35] | 1.09 [0.66, 1.78] | 1.22 [0.66, 2.27] | 3.11 [0.52, 18.58] |
|  | aRR2ǂ | Ref | 0.79 [0.43, 1.44] | 1.17 [0.71, 1.91] | 1.39 [0.76, 2.55] | 3.70 [0.56, 24.29] |
| NICU admission | aRRł | Ref | 0.91 [0.54, 1.54] | 1.11 [0.72, 1.71] | 1.36 [0.77, 2.42] | 2.35 [0.33, 16.73] |
|  | aRR2ǂ | Ref | 0.91 [0.54, 1.55] | 1.15 [0.75, 1.77] | 1.42 [0.80, 2.51] | 2.42 [0.36, 16.22] |

*ARR (adjusted risk ratio), HTN (hypertension), PPH (postpartum hemorrhage), RR (risk ratio), SGA (small for gestational age, birthweight <10^th^ centile for gestation)*

ł *- adjusted for maternal age, body mass index at booking, ethnicity, parity, smoking status, alcohol use, previous pre-eclampsia and previous gestational diabetes using imputation.*

ǂ- *adjusted for maternal age, body mass index at booking, ethnicity, parity, smoking status, alcohol use, previous pre-eclampsia and previous gestational diabetes not using imputation.*

NAǁ - no events in this group

**Table S2:** Sensitivity analysis restricted to study visit BP measurements: diagnostic test properties of ACC/AHA BP categories for detection of adverse pregnancy outcomes, according to ACC/AHA BP categories as thresholds for abnormal BP

| **Outcomes and BP thresholds** | **Sensitivity (95% CI)** | **Specificity (95% CI)** | **Positive LR (95% CI)** | **Negative LR (95% CI)** |
| --- | --- | --- | --- | --- |
| **Preterm Birth** | | | | |
| ‘Elevated BP’ | 0.69 (0.59, 0.78) | 0.33 (0.31, 0.36) | 1.03 (0.90, 1.19) | 0.93 (0.69, 1.26) |
| ‘Stage 1 HTN’ | 0.46 (0.36, 0.57) | 0.54 (0.51, 0.57) | 1.01 (0.81, 1.26) | 0.99 (0.82, 1.20) |
| ‘Non-severe ‘Stage 2 HTN’ | 0.13 (0.07, 0.21) | 0.88 (0.87, 0.90) | 1.13 (0.67, 1.91) | 0.98 (0.91, 1.06) |
| ‘Severe ‘Stage 2 HTN’ | 0.02 (0.00, 0.07) | 1.00 (0.99, 1.00) | 9.57 (1.62, 56.6) | 0.98 (0.95, 1.01) |
| **Postpartum haemorrhage** | | | | |
| ‘Elevated BP’ | 0.72 (0.65, 0.78) | 0.34 (0.31, 0.37) | 1.08 (0.98, 1.19) | 0.84 (0.67, 1.06) |
| ‘Stage 1 HTN’ | 0.54 (0.47, 0.62) | 0.55 (0.52, 0.58) | 1.21 (1.05, 1.39) | 0.83 (0.70, 0.97) |
| ‘Non-severe ‘Stage 2 HTN’ | 0.14 (0.09, 0.19) | 0.88 (0.87, 0.90) | 1.17 (0.80, 1.72) | 0.98 (0.92, 1.04) |
| ‘Severe ‘Stage 2 HTN’ | 0.00 (0.00, 0.02) | 1.00 (0.99, 1.00) | - | 1.00 (1.00, 1.01) |
| **Small-for-gestational age** | | | | |
| ‘Elevated BP’ | 0.69 (0.59, 0.78) | 0.34 (0.31, 0.36) | 1.04 (0.90, 1.20) | 0.92 (0.67, 1.25) |
| ‘Stage 1 HTN’ | 0.53 (0.43, 0.64) | 0.54 (0.52, 0.57) | 1.17 (0.96, 1.42) | 0.86 (0.69, 1.07) |
| ‘Non-severe ‘Stage 2 HTN’ | 0.16 (0.09, 0.25) | 0.89 (0.87, 0.90) | 1.39 (0.86, 2.27) | 0.95 (0.87, 1.04) |
| ‘Severe ‘Stage 2 HTN’ | 0.01 (0.00, 0.06) | 1.00 (0.99, 1.00) | 3.79 (0.43, 33.55) | 0.99 (0.97, 1.01) |
| **NICU admission** | | | | |
| ‘Elevated BP’ | 0.69 (0.60, 0.77) | 0.34 (0.31, 0.36) | 1.04 (0.92, 1.18) | 0.92 (0.70, 1.21) |
| ‘Stage 1 HTN’ | 0.52 (0.42, 0.61) | 0.54 (0.52, 0.57) | 1.14 (0.95, 1.36) | 0.89 (0.73, 1.07) |
| ‘Non-severe ‘Stage 2 HTN’ | 0.16 (0.10, 0.24) | 0.89 (0.87, 0.90) | 1.39 (0.90, 2.16) | 0.95 (0.88, 1.03) |
| ‘Severe ‘Stage 2 HTN’ | 0.01 (0.00, 0.05) | 1.00 (0.99, 1.00) | 2.92 (0.33, 25.89) | 0.99 (0.98, 1.01) |

*HTN (hypertension), CI (confidence interval) LR (likelihood ratio) NICU (Neonatal intensive care unit)*
